# Selective Hydrogenation of 1-Methylnaphthalene to 1-Methyldecalins with Supported Ni Catalysts for Hydrogen Storage: The Influence of the Nature of the Support

**DOI:** 10.3390/molecules31111782

**Published:** 2026-05-22

**Authors:** Anastasiya Shesterkina, Valeriya Myakota, Petr Pribytkov, Sergey Dunaev, Gennady Kapustin, Igor Mishin, Natalya Gordeeva, Leonid Kustov, Alexander Kustov

**Affiliations:** 1 Chemistry Department, Lomonosov Moscow State University, Leninskie Gory 1/3, 119991 Moscow, Russia; pribytkov_@mail.ru (P.P.); sfdunaev@mail.ru (S.D.); lmkustov@mail.ru (L.K.); 2 N.D. Zelinsky Institute of Organic Chemistry of the Russian Academy of Sciences, Leninsky Avenue 47, 119991 Moscow, Russia; leram01@list.ru (V.M.); gik@ioc.ac.ru (G.K.); igormish42@gmail.com (I.M.); natusya1320@gmail.com (N.G.)

**Keywords:** hydrogenation, 1-methylnaphthalene, nickel catalysts, hydrogen storage

## Abstract

1-Methylnaphthalene and the products of hydrogenation exhibit a high hydrogen storage capacity (6.6 wt.%), which makes them extremely promising as liquid organic hydrogen carriers. In this work, effective monometallic catalysts, 15Ni/Al_2_O_3_, 15Ni/Al_2_O_3_-SiO_2_, and 15Ni/Sib-ox, were synthesized and first investigated for hydrogenation of 1-methylnaphthalene to 1-methyldecalins. The prepared catalysts were characterized using a set of physicochemical analysis methods: SEM-EDX, TEM, XRD, N_2_ adsorption–desorption, FTIR and UV-vis-DRS. The catalytic activity of the samples in the hydrogenation reaction of 1-methylnaphthalene (100 min, 4 MPa, 240 °C) was studied in comparison to the traditional catalyst of hydrogenation, Ni Raney. The 15%Ni/Sib-ox catalyst showed a 100% conversion and high selectivity of 85.2% with respect to the target product 1-methyldecalins, while in the presence of Ni Raney, a selectivity of 74.3% was achieved with complete conversion of the substrate.

## 1. Introduction

One of the main problems associated with environmental pollution is the emissions of exhaust gases generated during the combustion of petroleum-based fuels [1,2,3]. The intensive use of fossil fuels leads to serious negative consequences, such as the release of greenhouse gases into the atmosphere, which can cause global warming [4]. One of the ways to solve this problem is using renewable and environmentally friendly energy sources. Today, hydrogen fuel is recognized as one of the environmentally friendly alternatives to petroleum fuel [5,6].

It has a number of advantages: water is the only product of hydrogen oxidation, with no other by-products formed, hydrogen engines have simple designs and hydrogen offers advantages over gasoline engines in terms of fuel consumption. However, for all its advantages, this type of fuel has its drawbacks, mainly related to explosion hazards. Therefore, an important condition for its application is the development of safe gas storage systems.

For the safe storage of hydrogen, renewable systems based on liquid organic hydrogen carriers (LOHCs) can be used, which are capable of catalytic hydrogenation–dehydrogenation reactions. LOHCs are potentially cheap, safe, and easy to handle [7]. In addition, they provide long-term energy storage without evaporation or other loss of hydrogen, and they are also compatible with transport infrastructure. This allows the gradual implementation of this technology and provides an opportunity for the oil industry to reduce carbon dioxide emissions.

Systems based on LOHCs have long attracted the attention of researchers, and interest in them is increasing every year [8,9,10,11,12]. For example, Crabtree [13] proposed the use of organic heterocycles as LOHCs, providing safe storage and simple temperature control. Potential candidates for LOHCs have also been analyzed for various applications such as energy storage in buildings, energy transportation, and fuel use in transport [14,15,16,17,18,19]. 

A common system of liquid organic hydrogen carriers is the naphthalene–decalin system, which has a hydrogen capacity of 7.2 wt.% [20]. Despite its high hydrogen capacity, naphthalene is a solid under normal conditions, which imposes a number of restrictions related to both the hydrogenation and dehydrogenation processes. The design of new hydrogen storage systems has become a priority. In our previous work [21], a comparative analysis of catalysts based on noble and non-noble metals in the hydrogenation of naphthalene and its methyl derivatives was carried out. It should be noted that hydrogenation reactions involving 1-methylnaphthalene and 2-methylnaphthalene have been poorly studied. At the same time, 1-methylnaphthalene is considered the most promising substrate in terms of its use as a fuel additive or as an independent energy carrier, since its melting temperature is −20 °C and under normal conditions, it is a liquid substance, while 2-methylnaphthalene is a solid [22].

A significant part of the research on the hydrogenation of these aromatic compounds is devoted to the use of catalysts based on precious metals [23,24,25,26,27], but the use of traditional hydrogenation catalysts is limited due to the high cost of compounds based on platinum group metals. Catalysts containing non-noble metals have a significant cost advantage over catalysts based on noble metals [28]. Such systems often have distinctive catalytic characteristics and reactivity. For example, nickel catalysts obtained by co-impregnation with polyols and deposited on an MCM-41 carrier were studied in the naphthalene hydrogenation reaction [29]. The activity of the hydrogenation catalysts was very high, with a 100% conversion at a low temperature of 55 °C and a tetralin selectivity of 93.9%. Other studies [30,31] have investigated heterostructured catalysts based on Ni-NiO in the selective hydrogenation of 2-methylnaphthalene. The results showed that when using NiO/Al_2_O_3_-370R, the conversion of 2-methylnaphthalene was 90.8%, and the yield of the target product was 59.7%; however, to achieve such indicators, harsh reaction conditions of 4 MPa H_2_ and 340 °C for 8 h are required. At the same time, the development of new selective hydrogenation catalysts based on Ni nanoparticles as a replacement for expensive platinum catalysts is an urgent task, due to their attractive cost–effectiveness ratio and their ability to activate hydrogen under mild conditions.

The performance of nickel catalysts can be significantly improved by the choice of support, catalyst preparation method or metal loading [21]. It is worth noting that in the hydrogenation of various liquid organic hydrogen carriers, active carbon [32], SiO_2_ [33], SiO_2_–Al_2_O_3_ [34], Al_2_O_3_ [35], and zeolites [36] are often used as carriers. Using carbon materials with a neutral or mildly alkaline surface as supports for Ni catalysts generally helps minimize the occurrence of hydrogenation side reactions. Additionally, the developed porous surface facilitates the access of the reagent to the active metal. However, high chemical inertness does not enhance the potential of the metal stabilized on their surface. A solution to this problem may be to improve the efficiency of hydrogenation catalysts by increasing the functionalization of the surface of the carrier. A characteristic of modern nanostructured carbon carriers, such as sibunit or carbon nanotubes, is the ability to control surface chemistry through modification. This, unlike conventional activated carbons, allows for the creation of the desired microstructure of these carriers. At the same time, with regard to carbon materials, it is known that oxidative surface treatment with nitric acid can lead to the formation of defective areas and greater surface functionalization, which in turn contribute to the fixation of the metal mainly on the outer surface [37,38].

Thus, the purpose of this work is to study the catalytic activity of nickel catalysts supported on various carriers, Al_2_O_3_, Al_2_O_3_-SiO_2_ and oxidized sibunit, in the hydrogenation reaction of 1-methylnaphthalene and compare the results obtained with the catalytic activity of the commercial Ni Raney catalyst.

## 2. Results and Discussion

### 2.1. Physicochemical Properties of the Catalysts

**Figure 1:**
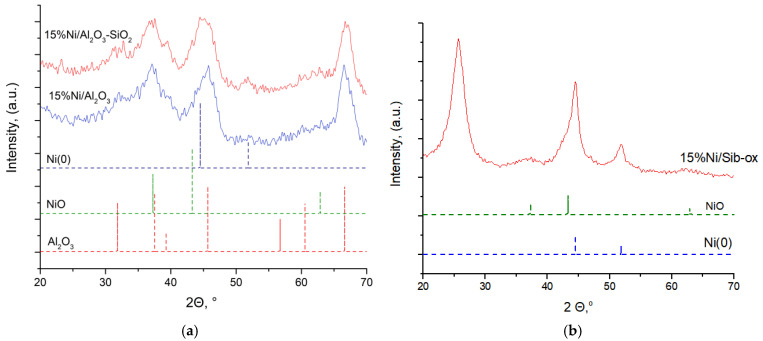
XRD patterns of Ni catalysts: Al_2_O_3_-supported catalysts (**a**) and sibunit-supported catalyst (**b**).

The phase composition of the obtained catalysts was investigated using the XRD method (Figure 1). For the 15Ni/Sib-ox catalyst, one of the most pronounced reflections is observed at 26° with a weak intensity at 43°, belonging to the sibunit phase. At the same time, reflections related to Al_2_O_3_ [PDF #50-0741] are noticeable on the diffractograms of samples 15Ni/Al_2_O_3_ and 15Ni/Al_2_O_3_-SiO_2_. For the Al_2_O_3_-supported catalysts, low-intensity reflections at 44.5° and 51.8° can be observed, corresponding to the peak positions of (111) and (200) planes of the FCC lattice of Ni^0^ [PDF #4-850] and low-intensity reflections at 43.2° and 62.8°, which relate to the nickel oxide phase NiO [PDF #47-1049]. In turn, reactions related to Ni^0^ and NiO are also observed in the sample supported on sibunit; in this case, the reactions of the active phase are more intense (Figure 1b). Based on the XRD data obtained, it can be concluded that the reduction temperature at 350 °C in a hydrogen flow does not allow the oxide phase to be completely restored to Ni^0^.

**Figure 2:**
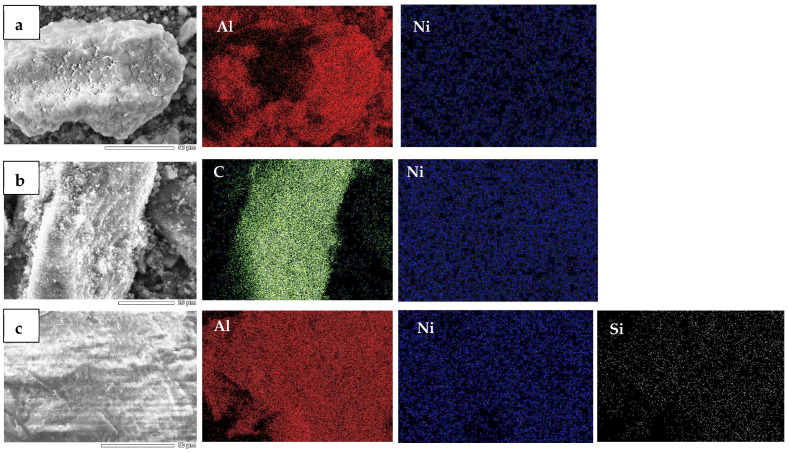
SEM images of Ni catalysts: (**a**) 15Ni/Al_2_O_3_, (**b**) 15Ni/Sib-ox, (**c**) 15Ni/Al_2_O_3_-SiO_2_.

The morphological characteristics of the supported nickel catalysts were studied via SEM-EDX and TEM methods. Figure 2 shows the elemental mapping of Ni-containing catalysts using SEM-EDX analysis. The obtained images show that Ni particles are uniformly distributed across the surface of all supports.

Energy dispersion analysis (Table 1) shows that the experimentally obtained mass content of nickel on the surface of the samples significantly differs from the theoretically set value during sample synthesis. The reason for this difference is probably that nickel deposition by impregnation occurs mainly on the inner surface of the carrier, which is unavailable for analysis by the SEM-EDX method. However, most of the available metal on the outer surface of the support, with the same method of sample synthesis, was found on a sample supported on Sib-ox.

ICP-OES was employed to quantify metal loading in prepared catalysts to check the % metal content. Table 1 presents the measured metal percentage for various catalyst samples. From the results obtained, it can be seen that the actual values obtained differ from the theoretically calculated values during the synthesis of catalysts. The deviations from the nominal values may be attributed to losses during the impregnation, drying, and calcination steps.

**Figure 3:**
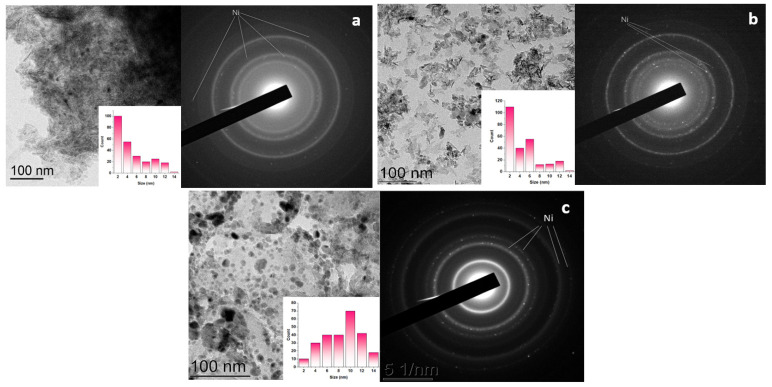
TEM patterns of Ni catalysts: (**a**) 15Ni/Al_2_O_3_, (**b**) 15Ni/Al_2_O_3_-SiO_2_, (**c**)15Ni/Sib-ox.

To further study the effect of the support nature on metal dispersion, nickel catalysts were analyzed using transmission electron microscopy (TEM), as shown in Figure 3. In TEM micrographs of catalysts supported on alumina and aluminosilicate supports, one can see the carriers’ needle-like nanoparticles. The average diameter of spherical nanoparticles in these samples is 2–6 nm. Additionally, a small number of large crystallites with a size of 10–13 nm can also be seen. In the sample supported on Sib-ox, a clearly defined pyrocarbon matrix consisting of nano-sized particles contiguous or connected to each other, with an almost spherical shape, was found, while the metal particles are distributed on both the external and internal surfaces of the carrier. The average diameter of spherical nanoparticles in this sample is 10–13 nm, and larger particle agglomerates (13–20 nm) are also visible. It can also be seen that all the samples contain a crystalline phase of metallic nickel.

Analysis of the BET specific surface area of the samples showed a decrease in the S_BET_ value for all monometallic catalysts along with a decrease in the volume of micro- and mesopores (Table 2). At the same time, for the catalyst supported on Sib-ox, a 2-fold decrease in the surface area is observed, which may be due to the predominant deposition of nickel on the outer surface of support; this is also consistent with the results of the SEM of this sample. The reduction in the pore size is probably also due to partial filling and blocking of the pores of the catalyst, which is typical for metal deposition by impregnation.

**Figure 4:**
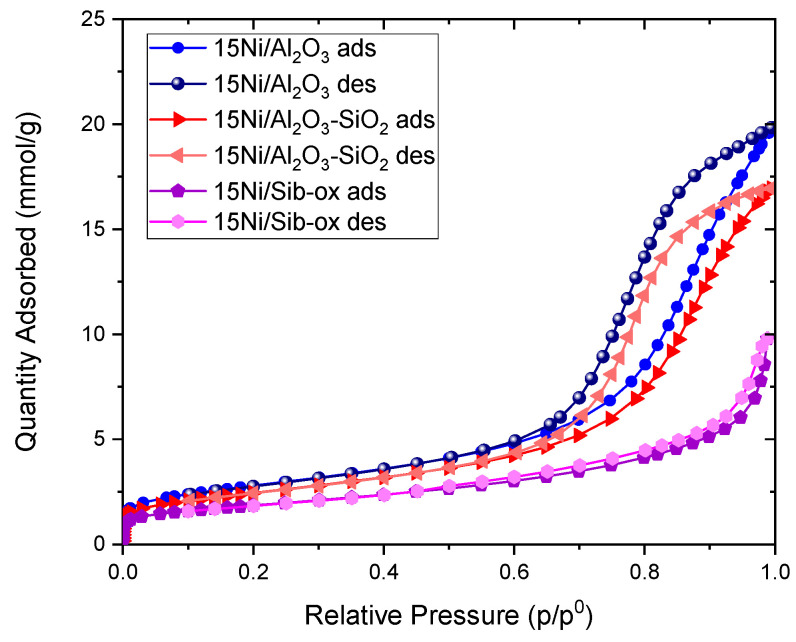
N_2_ adsorption–desorption isotherms of prepared catalysts.

Using N_2_ adsorption–desorption analysis, it was shown that the catalysts exhibit type IV adsorption isotherms, which are characteristic of materials with a mesoporous structure (Figure 4) [39]. On the aluminum oxide and aluminosilicate-based catalyst, there is a significantly increased amount of adsorbable N_2_ at a relative pressure of 0.99. This is due to the presence of much wider pores in the Al_2_O_3_ and Al_2_O_3_-SiO_2_ substrates compared to the oxidized sibunit.

**Figure 5:**
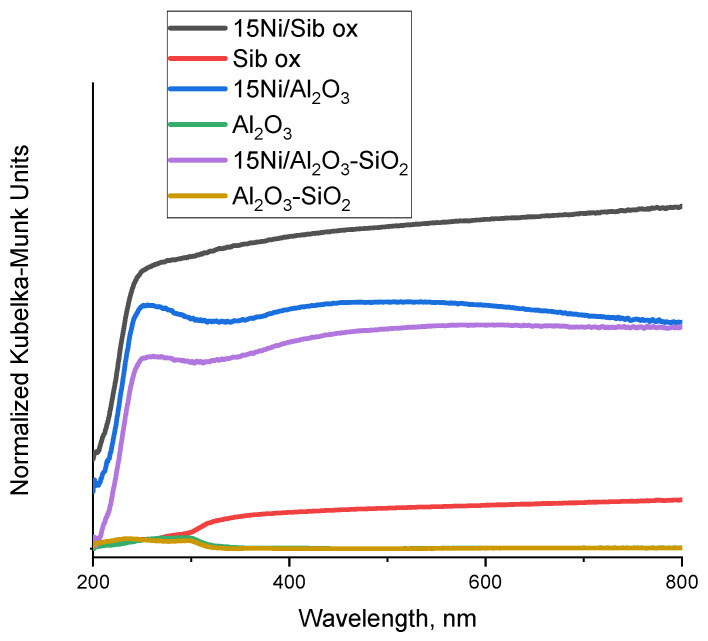
UV/VIS-DRS spectra of supports and Ni catalysts.

To identify the nickel species, the prepared Ni-containing catalysts were investigated via UV/VIS diffuse-reflectance spectroscopy. The spectra of the 15Ni/support samples are shown in Figure 5. The 15Ni/Al_2_O_3_ and 15Ni/Al_2_O_3_-SiO_2_ samples exhibit a broad UV band in the 230–330 nm region, which corresponds to the O^2−^ → Ni^2+^ charge transfer in the octahedral coordination of NiO [40,41]. The 15Ni/Sib-ox sample also shows this UV band, but to a lesser extent. In addition, the 15Ni/Al_2_O_3_ and 15Ni/Al_2_O_3_-SiO_2_ samples have a weak band at 390–420 nm. This indicates the formation of a chemical bond between nickel ions and the support. The appearance of a band in this region depends on the coordination, aggregation of Ni^2+^, and degree of dispersion, which indirectly indicates a stronger metal–carrier interaction between smaller Ni particles and the support [42].

**Figure 6:**
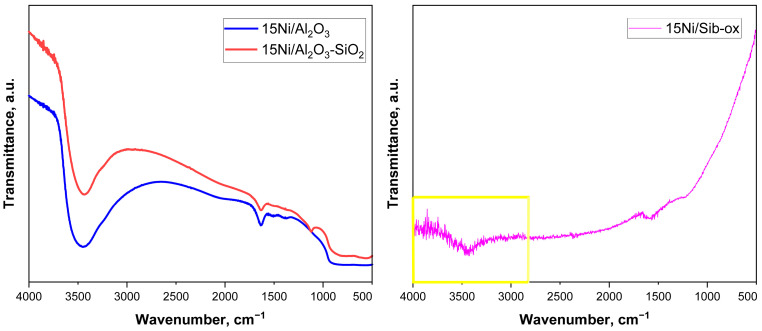
FTIR spectra supported Ni catalysts.

Figure 6 illustrates FTIR spectra of the 15Ni catalysts supported on different supports. The FTIR spectra for the samples of 15Ni/Al_2_O_3_ and 15Ni/Al_2_O_3_-SiO_2_ have a similar position, while the spectra for the 15Ni/Sib-ox catalyst have a different character. The broad absorption band in the range of 3600–3200 cm^−1^ and the band at 1637 cm^−1^ correspond to the OH stretching vibrations of adsorbed water and silanol groups, and bending vibrations of –OH groups, respectively, observed for all the samples [43]. The FTIR spectra of the 15Ni/Al_2_O_3_-SiO_2_ samples show an absorption band at 1105 cm^−1^ assigned to Si-O-Si asymmetric stretching vibrations [44,45]. The weak peaks at about 830 cm^−1^ and 560 cm^−1^ are ascribed to Ni–O–Ni and Ni–O–H stretching modes [46].

### 2.2. Hydrogenation of 1-Methylnaphthalene

**Figure 7:**
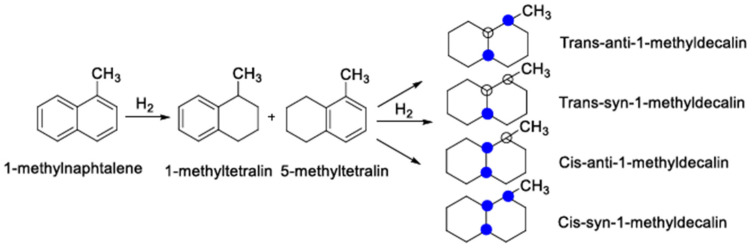
Scheme of hydrogenation of 1-methylnaphthalene [21].

All the obtained Ni catalysts were reduced in a hydrogen flow at 350 °C for 2 h before studying the catalytic activity in hydrogenation of 1-methylnaphthalene. Hydrogenation of 1-methylnaphthalene proceeds with the formation of two isomers of methyltetralins (1-methyltetralin and 5-methyltetralin) at the first stage and four isomers of methyldecalin at the second stage. 1-methyldecalin has four stereoisomers: trans-anti-, trans-syn-, cis-anti-, and cis-syn-1-methyldecalin (Figure 7). Among the isomers of 1-methyldecalin, trans-anti-1-methyldecalin has the highest thermodynamic stability [47].

**Figure 8:**
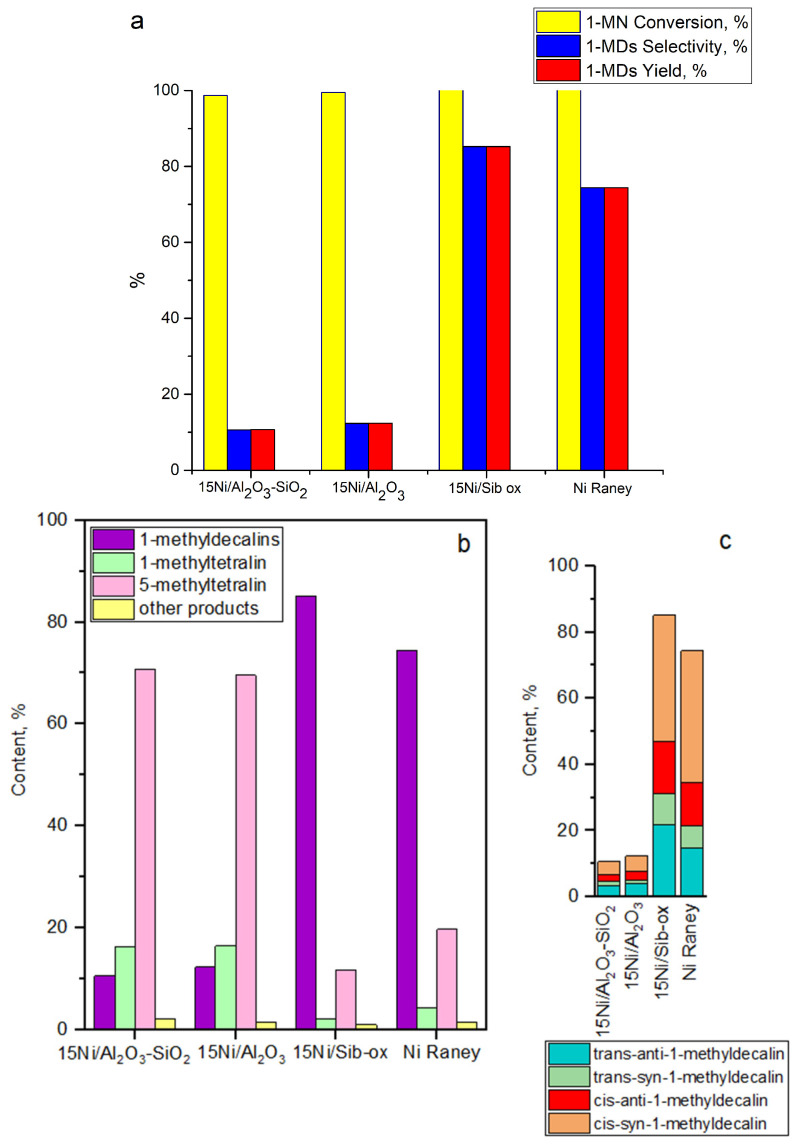
The catalytic activity of Ni catalysts in the hydrogenation of 1-methylnaphthalene to 1-methyldecalin: (**a**) conversion, selectivity and yield, (**b**) the distribution of reaction products, (**c**) the distribution of isomers of 1-methyldecalin.

The results of the catalytic hydrogenation of 1-methylnaphthalene to 1-methyldecalin are shown in Figure 8. During the study, it was shown that all samples showed complete conversion during the reaction time (Figure 8a). The distribution of both reaction products and 1-methyldecalin isomers differs for all samples. It should be noted that during the hydrogenation of 1-methylnaphthalene, trace amounts of four isomers of 2-methyldecalin were observed due to transalkylation reactions, which were also observed earlier [48]. As can be seen in Figure 8b, on the samples 15Ni/Al_2_O_3_-SiO_2_ and 15Ni/Al_2_O_3_, intermediates of hydrogenation of 1-methylnaphthalene, methyltetralins with a clear predominance of 5-methyltetralin, are formed. This may be due to the smaller size of Ni particles, which are mainly fixed in the pores of the carrier, reducing their accessibility to the substrate and making hydrogenation ineffective.

The composition of the products obtained on the 15Ni/Sib-ox and Ni Raney samples is significantly different from the samples supported on alumina and aluminosilicate; the main products were 1-methyldecalins. The 15%Ni/Sib-ox catalyst turned out to be even more selective with respect to 1-MD target products than Ni Raney. Such high rates may be associated with the predominant deposition of nickel on the outer surface of the Sib-ox support, which is consistent with the SEM-EDX data, while in the case of Al_2_O_3_-based carriers, nickel deposition occurs mainly in the pores of the supports. Analysis of the composition of 1-methyldecalin isomers (Figure 8c) showed that during the reaction on all catalysts, cis-syn-1-methyldecalin prevailed among cis-1-methyldecalin isomers, and trans-anti-1-methyldecalin dominated among trans-1-methyldecalin isomers, aligning with the high thermodynamic stability of this stereoisomer.

**Figure 9:**
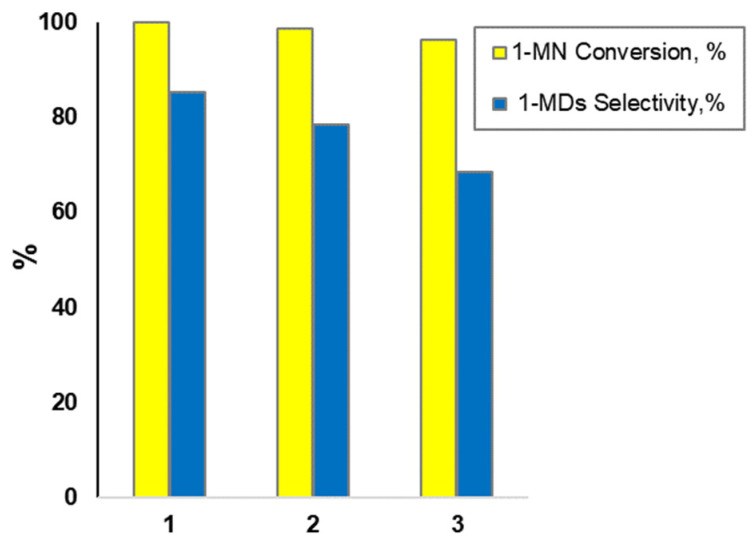
Stability of the 15Ni/Sib-ox catalyst in the hydrogenation of 1-methylnaphthalene to 1-methyldecalin.

The 15Ni/Sib-ox catalyst, which showed the best catalytic properties, was investigated for the possibility of reuse during three cycles (Figure 9). According to the data obtained, the catalyst without intermediate activation after each cycle showed high activity with a decrease in selectivity for 1-methyldecalins from 85.2% to 68.5%.

A comparison of the activity of various catalysts in the hydrogenation of 1-methylnaphthalene with published data is presented in Table 3. Thus, in experiments with a short contact time, a fairly high conversion of 1-methylnaphthalene was achieved, but the yield of 1-methyldecalins was low [36,49]. It should also be noted that platinum catalysts were tested [49], which did not demonstrate high activity. In another study [50], a similar trend was observed with a high conversion and low yield of the complete hydrogenation product, while reactions were carried out under more severe conditions (5 MPa, 350 °C) compared with our work (4 MPa, 240 °C). Thus, our work shows that nickel-based catalysts are highly effective in the hydrogenation reaction of 1-methylnaphthalene and can serve as a competitive alternative to noble metal-based catalysts.

## 3. Materials and Methods

### 3.1. Catalyst Preparation

The supported nickel catalysts were prepared using the incipient wetness impregnation method. The commercial oxides Al_2_O_3_ (S_BET_ = 250 m^2^/g, Saint-Gobain, Paris, La Defense, France) and Al_2_O_3_-SiO_2_ (S_BET_ = 225 m^2^/g, Saint-Gobain, Paris, La Defense, France) and oxidized sibunit named Sib-ox (S_BET_ = 240 m^2^/g, Paris, La Defense, France) were prepared according to the method of [37] and used as supports. Initially, Al_2_O_3_ and Al_2_O_3_-SiO_2_ were pre-calcined for 3 h at a temperature of 400 °C, and Sib-ox was dried for 2 h at a temperature of 110 °C. A support sample with a fraction of 0.25–0.5 mm was impregnated with the required volume of an aqueous solution of the Ni precursor (Ni(NO_3_)_2_∙6H_2_O, Acros Organic, Geel, Belgium). The impregnated catalysts were dried for 12 h at 100 °C, calcined in air for 3 h at 300 °C and then reduced for 2 h at 350 °C in a hydrogen flow (30 mL/min) with a heating rate of 2 °C/min. The mass content of nickel in all samples is 15 wt.%. Thus, monometallic Ni-containing catalysts of the composition 15Ni/Al_2_O_3_, 15Ni/Al_2_O_3_-SiO_2_, 15Ni/Sib-ox and commercial Ni Raney (Acros Organics, 50% slurry in water) were used to compare the catalytic activity of the obtained samples in the hydrogenation of 1-methylnaphthalene (98%, Component Reagent, Moscow, Russia) to methyldecalins.

### 3.2. Catalyst Characterization

The phase composition of the catalysts and the size of the obtained phases were determined by X-ray diffraction using an ARLX’TRA (Thermo Fisher Scientific, Waltham, MA, USA) diffractometer with nickel-filtered CuKa radiation (40 mA, 40 kV) with a scanning speed of 1.2° per minute in the scanning range of 20 < 2θ < 70°.

The nickel content was determined via inductively coupled plasma spectrometry (ICP-OES). Analysis was performed using an Agilent 720 instrument (Agilent Technologies, Santa Clara, CA, USA). A 25 mg portion of the solid sample was placed into 50 mL polypropylene Falcon tubes, and 15 mL of concentrated nitric acid and 5 mL of concentrated hydrochloric acid were added. All reagents were of chemically pure grade. The mixture was kept at 60 °C for 1 h and then left at room temperature for 12 h. The resulting solution was transferred to a 25.0 mL class B volumetric flask and diluted to the mark with deionized water. Before analysis, the solutions were diluted 10- and 100-fold. To enhance the reproducibility of the results, the Sc line at 335.372 nm was utilized as an internal standard at a concentration of 20 mg/L and was mixed with the sample.

The microstructure and morphological characteristics of the reduced samples were analyzed using scanning electron microscopy (SEM) on an SM40 electron microscope (Melytec, Hangzhou, China) equipped with an electron gun on a Schottky-type thermopolar cathode. The device allows you to set the energy of electrons in the range of 0.5 kV–30 kV. The elemental composition was determined using an Xplore 30 energy dispersion detector (Oxford Instruments, Abingdon, UK), with an X-ray photon energy resolution of 127 eV.

The morphology of the studied samples and the particle size distribution were studied using a transmission electron microscope (TEM) JEOL JEM-2100 (JEOL Ltd., Akishima, Japan). Before the measurements, the samples were deposited on carbon meshes from suspension in isopropanol. The images were obtained in the TEM mode of a bright field at an accelerating voltage of 200 kV. The average particle size was calculated by analyzing 5 micrographs with a sample of 250 nanoparticles of various sizes and shapes using the Digimizer 6.5.1. program (MedCalc Software bv. Ostend, Belgium).

Textural analysis of the substrates was performed by measuring the adsorption–desorption isotherms of N_2_ at 77 K using the ASAP 2020 Plus instrument (Micromeritics, Norcross, GA, USA).

The valence states of Ni on the support surface were studied using diffuse reflectance UV-Vis spectroscopy on a Shimadzu UV-3600 Plus spectrophotometer (Shimadzu, Kyoto, Japan) with an ISR-603 integrating sphere. BaSO_4_ was used as a reference sample and sample diluent. Spectra were recorded in the wavelength range of 200–800 nm at room temperature (25 °C). The resulting spectra were processed using UVProbe software (version 2.3). The electronic states of the catalyst’s elements were investigated using the FTIR method with a Bruker ALPHA II spectrometer (Bruker Optik GmbH, Ettlingen, Germany). 

### 3.3. Catalyst Activity Test

The catalytic properties of the pre-reduced Ni catalysts were investigated in the liquid-phase hydrogenation of 1-methylnaphthalene with molecular hydrogen using the 25 mL stainless steel autoclave. In a typical experimental procedure, the 0.350 g catalyst was added to 4 mL of the solvent-free substrate of so n_syb_/n_Ni_ ratio was equal to 25. Reactions were carried out under a H_2_ pressure of 4 MPa and a temperature of 240 °C at a stirring rate of 700 rpm and a reaction duration of 100 min. Reaction products were analyzed via Chromatec 5000 Crystal GC (Nizhny Novgorod, Russia) using the CR-5 capillary column (30 m × 2.5 mm) and a flame-ionization detector (FID) with undecane as the internal standard method. The temperature of the column was 150 °C, and the temperature of the evaporator and detector was 240 °C. The volume of the analyzed sample was 0.1 µL.

The catalytic characteristics of the samples were characterized by the selectivity of formation of the target reaction products and substrate conversion.

The conversion of the substrate $\chi_{1-MN}$ was determined by the following formula:

$$\chi_{1-MN}=100-\frac{S_{1-MN}\cdot 100\%}{S_{total}}\;\left(\%\right),$$

where *S*_1−*MN*_ is the peak area of 1-methylnaphthalene and *S_total_* is the sum of the areas of all peaks.

The selectivity of the formation of reaction products (*S_prod_*) was determined by the following formula:

$$S_{prod}=\frac{C_{prod}\cdot 100\%}{\chi_{1-MN}}\;\left(\%\right),$$

where *C_prod_* is the percentage of the product in the mixture.

## 4. Conclusions

In this work, effective monometallic Ni catalysts were synthesized on various carriers: Al_2_O_3_, Al_2_O_3_-SiO_2_ and oxidized Sibunit (Sib-ox). The textural properties and morphological characteristics of these samples were characterized using a complex of physico-chemical methods: SEM-EDX, TEM, XRD, FTIR, UV/VIS-DRS and N_2_-adsorption. The synthesized catalysts were first investigated in the full hydrogenation reaction of 1-methylnaphthalene, which showed high efficiency under mild conditions. The 15%Ni/Sib-ox catalyst showed the best catalytic characteristics in the hydrogenation reaction of 1-methylnaphthalene (100 min, 4 MPa, 240 °C), demonstrating 100% conversion and high selectivity of 85.2% relative to the target product 1-methyldecalins. It is also worth noting that this result was obtained due to the preferential deposition of nickel on the outer surface of the Sib-ox carrier, which, in turn, contributed to a more complete reaction. Thus, the development of new hydrogenation catalysts based on non-noble metals as a replacement for expensive platinum catalysts is an urgent task in the field of heterogeneous catalysis.

## Figures and Tables

**Table 1:** Mass contents of Ni in the supported catalysts obtained by SEM-EDX and ICP-OES.

Sample	Theoretical Ni Content, wt.%	Surface Average Ni Content ^a^, wt.%	Ni Content ^b^, wt.%
15Ni/Al_2_O_3_	15	4.9	10.5
15Ni/Al_2_O_3_-SiO_2_	15	6.1	11.2
15Ni/Sib-ox	15	7.9	14.3

^a^—data based on SEM-EDX, ^b^—data based on ICP-OES.

**Table 2:** Structural properties of synthesized nickel samples and carriers.

Sample	S_BET_, m^2^/g	V_tot_, cm^3^/g	V_micro_, cm^3^/g	V_meso_, cm^3^/g	D_pores_, nm
Sib-ox	240	0.41	0.02	0.39	9
15Ni/Sib-ox	147	0.34	0.01	0.33	9
Al_2_O_3_	254	0.80	0.01	0.79	13
15Ni/Al_2_O_3_	223	0.69	0.01	0.68	12
Al_2_O_3_-SiO_2_	232	0.72	0.01	0.71	12
15Ni/Al_2_O_3_-SiO_2_	196	0.59	0.01	0.58	11

**Table 3:** Data on the catalytic properties of catalysts known in the literature and our catalysts in the 1-methylnaphthalene hydrogenation.

Catalyst	Reaction Conditions	Conversion, %	Yield 1-MDs, %	Ref.
8NiO/Al_2_O_3_ + ZSM-5	Atmospheric pressure, 200 °C, 100 mg, t = 0.5 s	94.3	1.4	[36]
1.5Pt/AlSBA-15	Atmospheric pressure, 240 °C, 250 mg, W/F = 0.8 g s/cm^3^	79.0	17.9	[49]
2Pt/AlSBA-15	5 MPa, 350 °C, WHSV = 2 h^−1^, 10 vol.% 1-MN in n-heptane, W/F = 0.8 g s/cm^3^, 250 mg	91.8	21.6	[50]
15Ni/Sib-ox	4 MPa, 240 °C, 100 min, 350 mg, liquid phase process	100	85.2	In this work

## Data Availability

Data will be made available on request.
